# Human Decidual NK Cells: Unique and Tightly Regulated Effector Functions in Healthy and Pathogen-Infected Pregnancies

**DOI:** 10.3389/fimmu.2013.00404

**Published:** 2013-11-25

**Authors:** Philippe Le Bouteiller

**Affiliations:** ^1^Institut National de la Santé et de la Recherche Médicale, UMR 1043, Toulouse, France; ^2^Centre National de la Recherche Scientifique, UMR 5282, Toulouse, France; ^3^Centre de Physiopathologie Toulouse Purpan, Université Paul Sabatier, Toulouse, France

**Keywords:** decidual NK cell, pregnancy, cytotoxicity, cytokine, angiogenic factors, cytomegalovirus, pathogens

## Abstract

NK cells present in the peripheral blood (PB) respond rapidly to pathogens or pathogen-infected cells by various means including cytotoxicity and release of cytokines and chemokines. In addition they modulate adaptive immunity via the interaction with dendritic cells. Decidual NK cells (dNK) are poorly cytotoxic in healthy pregnancy, both in humans and rodents, when compared to their PB counterparts. We will discuss recent findings that may contribute to answer the following questions: (i) Do dNK possess functional killing machinery in normal healthy pregnancy? (ii) If so, what are the regulatory mechanisms that negatively control this effector function? (iii) Have dNK from early pregnant uterus the intrinsic ability to kill pathogen-infected autologous maternal uterine cells and/or produce soluble factors that stimulate the anti-pathogen adaptive immune response? (iv) Do dNK undergo a receptor repertoire profile shift when they are in contact with pathogen-infected uterine cells? (v) Which pathogen-mediated signal(s) and molecular interactions subvert the inhibition of dNK cytolytic activity?

## Introduction

During the last decade, an increasing body of data based on genetic and functional studies have provided compelling evidence that during early healthy pregnancy, decidual NK (dNK) cells, the dominant lymphocyte population accumulated in the *decidua basalis* where the trophoblast cells infiltrate, exhibit unique functional and phenotypic characteristics (1–5). More than 95% of dNK are CD56^bright^ CD16^neg^ CD160^neg^ (6). As opposed to dNK, the major peripheral blood (PB)-NK cell subset is CD56^dim^ CD16^+^ CD160^+^, a specific phenotype associated with cytotoxic activity (6, 7). dNK cells express the two-domain killer immunoglobulin-like receptor (KIR) at higher frequency than PB-NK (8). This KIR2D repertoire is biased toward recognizing HLA-C, the dominant KIR ligand expressed by fetal extravillous trophoblast (8). PB-NK mostly contribute to the innate immunity against pathogen-infected or tumor cells by their cytolytic activity as well as release of pro-inflammatory cytokines and chemokines (9). In healthy pregnant uterus, dNK are poorly cytotoxic and have specialized pregnancy-specific functions, including a capacity to produce angiogenic molecules and to control trophoblast invasion (10–15). Until very recently (16), little information existed on the dNK capability of limiting local decidual pathogen infection.

## Decidual NK Cells Produce Soluble Factors That Influence Placental Development in Normal Pregnancy

Human dNK, in contrast with PB-NK, control two crucial functions in early healthy pregnancy. Firstly, dNK promote vascular growth in the decidua through the production of vascular endothelial growth factor (VEGF) and placental growth factor (PlGF) (9, 13, 17–19) as well as of angiopoietin 1, angiopoietin 2, and TGF-β1 (15, 20). The release of these proangiogenic factors by dNK cells depends on the engagement of both NKp30 and NKp44 activating receptors by their specific ligands present on stromal decidual cells and extravillous trophoblast (17). Engagement of another NK cell receptor, KIR2DL4, by soluble HLA-G specific ligand was shown to induce the production of proangiogenic cytokines (21). Since KIR2DL4 is expressed by dNK either at the cell surface (22) or intracellularly (21), and soluble HLA-G is secreted by trophoblast (23), this receptor-ligand couple is likely to contribute to the remodeling of the maternal vasculature in early pregnancy. The same authors have demonstrated that the KIR2DL4-induced senescence secretome of PB-NK can promote vascular remodeling and angiogenesis (24). Analysis of the dNK cell transcriptome revealed a strong similar senescence signature (24). Secondly, dNK release interleukin-8 (IL-8) and interferon-inducible protein-10 (IP-10) chemokines that favor the migration of the extravillous cytotrophoblast into the *decidua basalis* by interacting with the CXCR3 and CXCR1 receptors expressed by the trophoblast cells (17). Migration of extravillous trophoblast cells results in the invasion of spiral arteries thus contributing to the uterine vascular remodeling crucial for the placental development and outcome of pregnancy (25). Such production of chemoattractants by dNK is due to the engagement of NKp30 and NKp44 by their specific ligands expressed by trophoblast and stromal decidual cells (17). Similar interactions between dNK and trophoblast were observed in pregnant mouse, leading to mesometrial spiral artery remodeling (26–28). Activation of the KIR2DS1 dNK receptor by HLA-C2 was shown to produce the GM-CSF soluble factor, which enhanced migration of trophoblast (29). Other reports from our laboratory indicated that a specific engagement of NKp30 activating receptor on dNK induced the release of several soluble inflammatory and growth factors, including IFN-γ, TNF-α, MIP1-α, MIP1-β, and GM-CSF (22, 30). During viral infection, these molecules are important to recruit and activate eosinophils, macrophages, and dendritic cells (31).

## Do Decidual NK Cells Possess Functional Lytic Machinery in Normal Healthy Pregnancy?

Human PB-NK are important innate immune effector cells that rapidly respond to and destroy malignant or virally infected cells (32). PB-NK kill infected cells via the polarized release of cytotoxic granules and the formation of immune synapse between the target cell and the PB-NK. The balance between activating and inhibitory NK cell receptor engagement upon recognition of specific cellular ligands determines the degree of NK cell activation or inactivation. In contrast to PB-NK, it has been demonstrated that both human dNK and mouse uterine NK cells displayed a poor ability to kill various classical NK cell targets (12, 28, 33). dNK cells fail to polarize their microtubules organizing centers and perforin-containing granules to the synapse when they are in contact with HLA class I negative target cells (33). Although dNK cells fail to fully exert their lytic properties in normal pregnancy, they have lytic granules containing perforin, granzyme, and granulysin (34), indicating that they are potentially capable of cytolytic activity. Using redirected cell lysis assay, it has been shown that specific monoclonal antibody engagement of NKp46 activating receptor expressed on freshly isolated dNK induced intracellular calcium mobilization, granule exocytosis, and efficient P815 target cell lysis (22). It is of note that uterine NK cells in pregnant mice express the natural cytotoxicity receptor 1 (NCR1), an ortholog of human NKp46 also present in other species (35). This evolutionary conservation suggests that NKp46 could be involved in pathogen recognition (36). Experiments performed in mice homozygous for NCR1 loss revealed the role of NCR1 in the clearance of influenza virus infection (37). It has been demonstrated that granulysin release from mouse uterine NK cells in spontaneous abortion induced apoptosis of extravillous cytotrophoblast (28). All of these data strongly suggested that both human and mouse NK cells have the capability to exert a cytotoxic effector function in the pregnant uterus.

## What are the Regulatory Mechanisms That Negatively Control the Cytotoxic Function of Decidual NK Cells in Normal Pregnancy?

The importance of the balance between activating and inhibitory signals mediated by the specific engagement of activating and inhibitory NK cell receptors has been underlined by a number of reports (38). In addition to the inhibitory KIRs, three other dNK receptors play an inhibitory function, namely 2B4, CD94/NKG2A, and LILRB1/ILT2. Whereas 2B4 was shown to be expressed on all dNK (22, 39), both CD94/NKG2A and LILRB1/ILT2 are only present on a subset of dNK (8, 12, 22, 33). Specific interaction between inhibitory dNK receptors and either MHC class Ia (HLA-C) or MHC class Ib molecules (HLA-E, HLA-G) in human play an important role (40). We found that the co-engagement of CD94/NKG2A inhibitory receptor triggered a drastic inhibition of the cytolytic function of freshly isolated dNK from early *decidua basalis* (22). These results suggest that *in situ* the CD94/NKG2A receptor interaction with its HLA-E specific ligand expressed by trophoblast and other decidual cells is a dominant negative regulatory mechanism that likely prevents unwanted cytotoxicity toward non-infected fetal-derived trophoblast cells. Another inhibitory mechanism depends on the engagement of the LILRB1/ILT2 dNK receptor by HLA-G which blocks dNK cytotoxicity (41, 42). It was also demonstrated that VEGF-C released by dNK in early pregnancy induced up-regulation of TAP-1 in extravillous cytotrophoblast protecting them from cytolysis (13). Expression of the inhibitory form of 2B4 receptor on dNK also contributes to negatively regulate lytic function of dNK (39). Several regulatory mechanisms thus contribute to block the functional cytolytic machinery of dNK in normal pregnancy.

## Have Decidual NK Cells from Early Pregnant Uterus the Intrinsic Ability to Kill Pathogen-Infected Autologous Decidual Cells and Produce Soluble Factors That Stimulate the Anti-Pathogen Adaptive Immune Response?

A variety of pathogens, including human cytomegalovirus (hCMV) (10), human immunodeficiency virus 1 (HIV-1) (43), hepatitis C virus (44), toxoplasma (45), *Plasmodium falciparum* (46), bacteria in chorioamnionitis (10, 47) can infect the *decidua basalis* and potentially spread to the fetus through anchoring villi which are in contact with maternal blood of the intervillous space (23, 48). We will mainly focus on data dealing with hCMV which frequently infects decidua and has been detected in decidual stromal, macrophages, and endothelial cells (49). Areas of decidual hCMV infection were shown not to contain T cell accumulation, suggesting that, in addition to the humoral immunity (10), dNK could be another effector cell of the local anti-viral innate immunity preventing virus spread to the fetus. This hypothesis was recently validated by the observation that on biopsies of placental samples from hCMV termination, dNK have been identified in the vicinity of hCMV positive cells (16).

### Decidual NK cells fully exert their killing effector function when in contact with hCMV-infected autologous decidual fibroblasts

A recent report from our lab has demonstrated that dNK play a role in the control of decidual cell hCMV infection (16). When exposed to hCMV-infected decidual autologous fibroblasts, a cell type abundantly present in decidual tissue, dNK acquires major functional and phenotypic changes (16). As opposed to dNK cells in contact to uninfected autologous decidual fibroblast, dNK cells engage immune synapse with hCMV-infected autologous stromal cells, polarize their lytic machinery toward infected target cells and efficiently kill them (16). Thus, the co-culture of dNK from early gestation with infected autologous decidual cells clearly enhances their cytotoxic ability.

### Decidual NK cells modulate their cytokine and chemokines production when in contact with hCMV- or HIV-1-infected decidual fibroblasts

Co-culture of dNK from early gestation with hCMV-infected autologous fibroblast induced a significant increased secretion of VEGF-A, sICAM, CXCL-1, IL-6, Granzyme-B, and MCP-1 by comparison with dNK in contact with uninfected similar target cells (16). IL-6 is a multifunctional cytokine that includes directing T cells differentiation in adaptive immunity (50). Although the first trimester pregnancy decidua is permissive to HIV-1 infection *in vitro*, the frequency of early *in utero* mother-to-child transmission of HIV-1 is low, suggesting efficient inhibitory regulatory mechanisms (51). dNK secrete cytokines and chemokines known to inhibit HIV-1 infection like CCL-3 and CCL4 or CXCL-12 (51). The killing of HIV-1-infected decidual cells by dNK could be mediated by specific engagement of NK cell receptors but also by induction of apoptosis mediated by the Fas/FasL pathway (51) as dNK were shown to produce FasL (52).

## Do Decidual NK Cells Undergo a Receptor Repertoire Profile Shift When They are in Contact with Pathogen-Infected Uterine Cells?

hCMV infection modulates dNK cell receptor repertoire ability to kill virally infected decidual cells. Such a killing requires interaction between dNK cell activating receptors and specific ligands expressed at the cell surface of autologous decidual infected cells. Co-culture of dNK for 48 h with hCMV-infected decidual fibroblasts and without addition of IL-2 or IL-15, significantly increased the number of CD56^dim^ dNK, associated with appearance of CD16 (16). This dNK phenotype is consistent with acquisition of a cytotoxic profile (16). A recent study similarly reported a significant increase of the CD56^dim^ CD16^+^ dNK subpopulation as early as 12 h after *in vitro* infection with *Toxoplasma* when compared to uninfected dNK (53). Furthermore, hCMV infection induced increased levels of expression of NKp44 and NKG2C receptors (16). Another study found that hCMV infection promoted a persistent expansion of PB-NK cells that express the CD94/NKG2C activating receptor (54). It is of note that the CD94-NKG2 system is highly conserved in both rodents and primates (55), highlighting the critical activating function of this receptor/ligand couple. No change of NKp30 expression was noticed on dNK cells after contact with hCMV-infected fibroblast (16). When comparison was made between dNK and PB-NK, no significant change was observed in the expression of PB-NK cell repertoire after contact with hCMV-infected cells (16).

Disappearance of the NKp46^high^ dNK subset and decrease of KIR2DL1, KIR2DL4, and LILRB1/ILT2 expression were also observed on dNK in contact with hCMV-infected decidual fibroblasts (16). Thus, exposure to hCMV-infected cells modulates dNK receptor repertoire. An up-regulation of the NKG2D activating receptor expression was reported in *Toxoplasma*-infected dNK which correlated with enhanced cytolytic activity against human extravillous cytotrophoblast target cells (53). Based on these data, infection of decidua by other pathogens is likely to trigger a change in the repertoire profile of dNK receptors.

## Which Pathogen-Mediated Signal(s) and Molecular Interactions Subvert the Inhibition of Decidual NK Cell Cytolytic Activity?

Using Fc-chimeric proteins, we found that hCMV infection of decidual fibroblast modulated the expression of several NKR ligands: NKp30L and NKG2DL were significantly increased whereas NKp44L and NKp46L decreased (16). Blocking the engagement of NKp44 with its specific ligand resulted in an increase killing of infected autologous fibroblast by dNK cells, suggesting the presence of an inhibitory form of NKp44. In contrast, blocking NKG2D ligation induced a decrease of dNK cytotoxicity. These observations suggested a crucial role of NKG2D-mediated cytotoxic function of dNK against hCMV-infected fibroblasts. Infection of decidual fibroblast resulted in a diminishment of HLA-E expression on their cell surface. Blockade of HLA-E ligation with its specific dNK cell receptor induced a decrease of dNK killing, suggesting that HLA-E on hCMV-infected autologous fibroblasts did bind the CD94/NKG2C activating receptor. In contrast, neither NKp46 nor NKp30 seemed to be implicated in dNK cell lytic response. dNK cell subset that expresses the LILRB1/ILT2 receptor could be involved in the control of HIV-1 infection as it has been demonstrated in the PB-NK counterpart (56).

All of these data, including those dealing with hCMV infection and summarized in Table 1, showed that pathogen-mediated signals (i.e., modulation of expression of dNK receptor-ligands systems) and molecular interactions (engagement of dNK receptors with specific pathogen-mediated ligands) contribute to subvert the inhibition of dNK cell cytotoxic activity observed in normal pregnancy.

**Table 1:** **Decidual NK receptor-ligands interactions, signals and cytotoxic effector functions**.

dNK co-culture with	dNK CD56 marker	dNK receptors	Decidual fibroblast ligands (L)	dNK cytotoxic signals	dNK cytotoxic effector function
Uninfected decidual fibroblasts	CD56^bright^ ∼95%	CD94/NKG2A^b^	HLA-E	Negative	NO
	CD56^dim^ ≤5%	KIR2DL1^b^	HLA-C	Negative	
		ILT2^b^	HLA-G^d^	Negative	
		2B4^a^		Negative	
		NKG2D^c^	NKG2D-L: high level	Negative	
		CD94/NKG2C	NKG2C-L	?	
		KIR2DL4^b^	HLA-G^d^	?	
		NKp30^a^	NKp30-L: low level	No signal	
		NKp44^a^	NKp44-L: high level	No signal	
		NKp46^c^	NKp46-L: low level	No signal	
		CD16^neg^			
hCMV-infected decidual fibroblasts	CD56^bright^ ∼48%	CD94/NKG2A: down	HLA-E: down	?	YES
	CD56^dim^ ∼40%	KIR2DL1: down	HLA-C	?	
		ILT2: down	HLA-G^d^	?	
		NKG2D^c^	NKG2D-L: down	Positive	
		CD94/NKG2C: up	HLA-E: down	Positive	
		KIR2DL4: down	HLA-G^d^	?	
		NKp30^a^	NKp30-L: up	No signal	
		NKp46: down	NKp46-L	No signal	
		NKp44: up	NKp44-L: down	Negative	
		CD16^b^			

^a^20–40% positive cells.

^b^50–80% positive cells.

^c^100% positive cells (flow cytometry).

^d^HLA-G is expressed on extravillous cytotrophoblast present among decidual adherent cells.

## Conclusion

Instead of being detrimental, dNK cells have positive effects in healthy human pregnancy as they promote placental development through the crucial release of angiogenic factors and attraction of extravillous trophoblast. After contact with hCMV-infected autologous decidual fibroblasts, dNK acquire cytotoxic capacity by subverting their killing inhibitory regulatory mechanisms occurring in normal pregnancy. Effector functions of dNK in pathogen-infected pregnancy require engagement of shifted repertoire of activating and/or inhibiting dNK cell receptors by specific ligands up- or down-regulated in pathogen-infected decidual cells, including HLA-E and NKG2DL. These results, if confirmed by further studies dealing with different viruses or pathogens, strongly suggest that, *in situ*, dNK are likely to contribute to local pathogen innate immune control and clearance during the initial phase of decidua infection.

## Conflict of Interest Statement

The author declares that the research was conducted in the absence of any commercial or financial relationships that could be construed as a potential conflict of interest.
